# The Clinical Impact of Flash Glucose Monitoring—a Digital Health App and Smartwatch Technology in Patients With Type 2 Diabetes: Scoping Review

**DOI:** 10.2196/42389

**Published:** 2023-03-15

**Authors:** Sergio Diez Alvarez, Antoni Fellas, Derek Santos, Dean Sculley, Katie Wynne, Shamasunder Acharya, Pooshan Navathe, Xavier Girones, Andrea Coda

**Affiliations:** 1 School of Medicine and Public Health College of Health, Medicine and Wellbeing University of Newcastle Newcastle Australia; 2 School of Health Sciences College of Health, Medicine and Wellbeing University of Newcastle Ourimbah Australia; 3 School of Health Sciences Queen Margaret University Edinburgh United Kingdom; 4 School of Biomedical Sciences and Pharmacy College of Health, Medicine and Wellbeing University of Newcastle Newcastle Australia; 5 Equity in Health and Wellbeing Research Program Hunter Medical Research Institute Newcastle Australia; 6 Department of Diabetes and Endocrinology Hunter New England Health John Hunter Hospital Newcastle Australia; 7 Central Queensland Hospital and Health Service Brisbane Australia; 8 Department of Research Universities de Catalunya Generalitat de Catalunya Barcelona, Cataluna Spain

**Keywords:** type 2 diabetes, flash glucose monitoring, digital health, smartwatch, scoping review, app, smartphone, mobile phone, mHealth, digital, application, technology, effective, management, glucose, monitoring, database, wearable, diabetes, diabetic, glucose monitoring

## Abstract

**Background:**

Type 2 diabetes has a growing prevalence and confers significant cost burden to the health care system, raising the urgent need for cost-effective and easily accessible solutions. The management of type 2 diabetes requires significant commitment from the patient, caregivers, and the treating team to optimize clinical outcomes and prevent complications. Technology and its implications for the management of type 2 diabetes is a nascent area of research. The impact of some of the more recent technological innovations in this space, such as continuous glucose monitoring, flash glucose monitoring, web-based applications, as well as smartphone- and smart watch–based interactive apps has received limited attention in the research literature.

**Objective:**

This scoping review aims to explore the literature available on type 2 diabetes, flash glucose monitoring, and digital health technology to improve diabetic clinical outcomes and inform future research in this area.

**Methods:**

A scoping review was undertaken by searching Ovid MEDLINE and CINAHL databases. A second search using all identified keywords and index terms was performed on Ovid MEDLINE (January 1966 to July 2021), EMBASE (January 1980 to July 2021), Cochrane Central Register of Controlled Trials (CENTRAL; the Cochrane Library, latest issue), CINAHL (from 1982), IEEE Xplore, ACM Digital Libraries, and Web of Science databases.

**Results:**

There were very few studies that have explored the use of mobile health and flash glucose monitoring in type 2 diabetes. These studies have explored somewhat disparate and limited areas of research, and there is a distinct lack of methodological rigor in this area of research. The 3 studies that met the inclusion criteria have addressed aspects of the proposed research question.

**Conclusions:**

This scoping review has highlighted the lack of research in this area, raising the opportunity for further research in this area, focusing on the clinical impact and feasibility of the use of multiple technologies, including flash glucose monitoring in the management of patients with type 2 diabetes.

## Introduction

### Overview

The rapid growth of easily accessible technology in the management of diabetes mellitus (DM) is undeniable, with the introduction of more sophisticated monitoring devices, including home-based self-monitoring glucometers [1], and more recently, continuous glucose monitoring (CGM) devices [2]. In the 2000s, real-time interstitial CGM (RT-iCGM) was introduced, although it still requires regular calibration (except for Dexcom G6 or G5 devices). A recent advance in commercial use is interstitial glucose monitoring through flash glucose monitoring (FGM) technology in the form of devices such as Abbott’s FreeStyle Libre [3,4].

As with most technological advances in DM, their impact is first noted in those patients who are most vulnerable for complications. Thus, typically RT-iCGM and FGM often find early clinical implementation in patients at high risk for complications, such as patients with type 1 DM and pregnancy [5]. More recent studies have supported RT-iCGM and FGM using cost-benefit models compared to self-monitoring glucometers in type 1 DM [6,7]. These technologies, often in conjunction with web-based analytic applications, have shown significant improvements in glycemic control during insulin initiation [8,9], routine care [10,11], and enhanced safety through the reduction of hypoglycemic events [12]. This has been particularly evident with severe hypoglycemia, variably defined in the literature as a blood glucose level ranging from <3.3 mmol/L to <2.8mmol/L [13].

In patients with type 2 DM, the use of RT-iCGM and FGM is less well defined [14]. In type 2 DM, the risk of hypoglycemia is related to the duration of diabetes and the use of hypoglycemic agents, particularly insulin [15]. Although the risk of hypoglycemia is considered to be lower than that in type 1 DM [16], the significantly higher rates of poor cardiovascular outcomes in the Action to Control Cardiovascular Risk in Diabetes Study Group (2008) and the Veterans Affairs Diabetes Trial [17] suggest that hypoglycemia in type 2 DM is not a benign phenomenon.

Evidence suggests that intensive insulin regimens in patients with type 2 DM carry the highest risk for severe hypoglycemia, with nocturnal hypoglycemia episodes having a particularly high burden of risk [18]. CGM has shown that hypoglycemia is more common than both the patient and their treating clinician anticipate [17]. A recent study of CGM showed 1.74 episodes per patient over a 5-day period, with 75% experiencing at least one asymptomatic episode and 64% of patients undergoing treatment modification as a result of the information gathered [19]. Closed-loop glucose monitoring technology has also been used in an inpatient type 2 DM setting to improve control without any increase in hypoglycemia [20-23].

A desired outcome of any glucose monitoring modality is the use of real-time data to promote positive behavior and therapeutic changes. Systems that provide immediate feedback to patients and decision support tools for patients and providers have demonstrated positive outcomes [24]. Furthermore, RT-iCGM and FGM provide additional information in the form of comprehensive data on the 24-hour glucose profile, current glucose trend, glucose variability, detection of periods of hypoglycemia and hyperglycemia, and estimated HbA_1c_ [25]. FGM has the additional advantage of factory calibration and interstitial blood sampling, thus avoiding the risk and discomfort of frequent subcutaneous sampling, significantly increasing its utility [26].

CGM or FGM usually consists of 3 components: a wearable sensor, a transmitter that wirelessly transmits glycemic data, and a receiver nearby that displays such readings to the user. This is further augmented by mobile health (mHealth) diabetic management systems. The World Health Organization defines mHealth as a medical and public health practice supported by mobile devices, such as mobile phones, patient monitoring devices, personal digital assistants, and other wireless devices. This may include proprietary commercial cloud-based app portals for the purpose of more complex analysis, such as glycemic trend analysis by either the patient, their treatment team, or an authorized carer [27].

A separate technological development is the sharp increase in both free and commercial mobile apps for the self-management of diabetes [28,29]. These apps are designed to assist patients in behavior change. Common features of these apps include the ability to track blood glucose, HbA_1c_, medications, physical activity, and body weight. Although apps for diabetes self-management can improve short-term outcomes, support from health care providers cannot be undervalued [30].

As a result of advances in information and communication technology, mobile phones and the internet technology are playing a growing role in interventions for health promotion and those aimed at preventing and managing diseases [31]. The largest burden of type 2 DM is not in high-income countries; given the high proportion of smartphone use in low- and middle-income countries, this could be a potential way of mitigating the small number of diabetes specialists in these regions.

With the most recent introduction of 5G networks, mHealth innovation continues to develop with the introduction of wearable devices [32]. One of the more easily accessible mHealth devices is the smartwatch with dominant global players such as Apple, Samsung, and Google expanding the market significantly. These wearable devices enable the application of smartwatches beyond traditional sectors [33,34] and their integration into daily management systems for diabetes.

### Aim and Rationale

The research question was identified from a preliminary scan of the literature and by drawing on the expertise of the research team and additional stakeholders. The scoping review aims to study the interface between 3 emerging technologies in the field of diabetes: (1) FGM, (2) app-based mHealth diabetic systems, and (3) smartwatch technology in the management of patients with type 2 DM.

In particular, the scoping review explores their combined impact as an integrated platform intervention on the clinical parameters of glycemic control and behavioral parameters relevant to self-management in type 2 DM.

Scoping reviews are particularly useful in emerging fields where it is still unclear what additional specific questions could be answered through a more precise systematic review [35] and can be used to map the key concepts underpinning the research area as well as to clarify the conceptual boundaries of a topic. Arksey and O’Malley [36] introduced the principle of scoping reviews as a mechanism for mapping the literature in a field of interest, providing a mechanism for the dissemination of research findings where a systematic review is not feasible due to the dearth of evidence in these emerging fields [36].

## Methods

### Types of Studies Included

To capture a comprehensive list of potential sources, a preliminary search of Ovid MEDLINE and CINAHL databases was performed to identify keywords and related subject headings in consultation with research librarians at University of Newcastle. Keywords were identified and combined to address the 4 components of the research question: (1) FGM, (2) mHealth-based health care delivery, (3) smartwatch technology, and (4) type 2 DM.

The initial search is then followed by an analysis of the text words contained in the title and abstract of retrieved papers and of the index terms used to describe the articles. A second search using all identified keywords and index terms was performed on Ovid MEDLINE (January 1966 to July 2021; Multimedia Appendix 1). This search strategy was adapted for EMBASE (January 1980 to July 2021), Cochrane Central Register of Controlled Trials (CENTRAL; the Cochrane Library, latest issue), CINAHL (from 1982), IEEE Xplore, ACM Digital Libraries, and Web of Science databases. No language or publication restrictions were applied. Reference lists of all included studies were checked for other potentially eligible papers that were searched in August 2021. All databases were then re-searched to ensure this review was updated to cover any recent titles and abstracts between July 2021 and July 2022.

Furthermore, ProQuest Dissertations and Theses Global (full text; 1997-present) were searched for relevant dissertations and theses; conference proceedings were searched via Scopus to capture any additional pertinent research, as this is an emerging field. Additional studies were identified by searching the reference lists of the included studies as well as the reference lists of related systematic reviews and meta-analyses. The rationale for including the breadth of literature formats is that, during a scan of the literature, a limited number of randomized controlled trials have evaluated the use of this combination of these technologies in the management of DM.

### Context

No restrictions will be placed on the types of settings in which the interventions have taken place, and as such, different study settings (eg, primary care, outpatient, inpatient, or community settings) will all be considered.

### Selection of Studies for Review

All search results were exported to Covidence systematic review management software (Veritas Health Innovation). Covidence is a web-based collaboration software platform that streamlines the production of systematic and scoping reviews. Two reviewers (AF and AC) independently searched the titles and abstracts of the retrieved literature via Covidence. Conflicts were resolved by a third reviewer (SDA) and through team consensus. Articles that met the inclusion criteria through abstract screening were reviewed in full. Both inclusion and exclusion criteria were revised in an iterative process as the search evolved, to best address the research question (Textbox 1).

**Inclusion and exclusion criteria.**

**Inclusion criteria**

- Diagnosis of type 2 diabetes mellitus at any age.
- mHealth interventions, including digital health apps and smartwatch technology.
- The following study types: randomized clinical trials, quasi-experimental, controlled before and after studies, and observation (eg, cohort, case-control, cross-sectional) studies.
- No language restrictions.

**Exclusion criteria**

- Type 1 and gestational diabetes mellitus.
- Conference abstracts or protocols only.
- If continuous glucose monitoring (CGM) or mHealth interventions could not be adequately separated and efficacy determined.

### Extraction of Results

A data extraction form was first prepared by SDA and AC. The data extraction process and assurance of the quality of data was iterative with frequent updates of the extraction form and the data collected from the studies. The data extraction process is shown in Table 1.

**Table 1:** Descriptive information for included studies.

Study	Population, sample, or context	Study design	Aim or objectives	Technologies used	Outcome measures	Results	Limitations
Kim et al [37]	29 adults with type 2 diabetes mellitus. Seoul National University Hospital, South Korea	12-week feasibility pilot study. One-arm group.	Test the feasibility of HbA_1c_ reduction using a patient-centered, smartphone-based, diabetes care system.	(1) Android-based app with four modules: glucose, diet, physical activity, and social network system; (2) Bluetooth glucometer; and (3) Bluetooth activity tracker	HbA_1c_, fasting plasma glucose, body weight, blood pressure, and various cholesterol measures (summary of diabetes self-care activities was used to evaluate the overall self-management activities for diabetes)	After 12 weeks participants had significantly decreased HbA_1c_ and FPGs. Reduction in HbA_1c_ were correlated with the number of daily glucometer inputs. Inputs were generally higher in older patients. Body weight and cholesterol measures were not statistically significant after 12 weeks.	No control group. Short observation time. Small sample size.
Shaw et al [38]	60 adults with type 2 diabetes mellitus. Southeastern United States.	6-month cohort prospective study.	To determine feasibility and acceptability of using multiple mHealth technologies in patients with T2DM^a^ and to also examine trajectories and patterns of diabetes-related variables.	(1) Glucometer “iHealth,” (2) Fitbit, (3) self-report mobile text messaging, and (4) cellular enabled scale by body trace	(1) Blood glucose; (2) physical activity—daily steps, distance travelled, and activity intensity; (3) medication adherence; and (4) weight	mHealth interventions not used to improve outcomes listed. Most used technology was the Fitbit. Participants who were younger had higher HbA_1c_ levels, and those who identified as Black were less likely to be engaged with their mHealth devices.	Only observational study; did not use control group for interventional impact. Small sample size.
Zahedani et al [39]	665 participants: healthy (448); prediabetic (25); and type 2 diabetic (192)	10-day observational study.	Investigating combined use of CGM^b^ and mobile app (Sugar AI) on glucose tracing, heart rate, and physical activity.	(1) Abbott FreeStyle Libre, (2) Xiaomi Mi Band 3 or Garmin watch, and (3) Sugar AI app	(1) Blood glucose, measured as time in range (TIR): 54-140 mg/dL for being healthy and prediabetes and 54-180 mg/dL for T2D^c^.	Authors concluded that a subgroup of those showing poor TIR (combined participants with T2DM and before diabetes) demonstrated an average of 22.7% improvement in TIR; 62.9% of participants with diabetes who showed an improved TIR had greater improvement in their daily variation.	Only observation study. Not randomized clinical trial. Short follow-up. Limited results provided on use of Garmin watch or MiBand 3 to improve outcome measures such as blood glucose levels or heart rate.

^a^T2DM: type 2 diabetes mellitus.

^b^CGM: continuous glucose monitoring.

^c^T2D: type 2 diabetes.

A descriptive-analytical narrative method was used to extract and chart the data from the selected articles [40]. Two reviewers (SDA and AF) independently collected the data using the extraction form. Charts were used to collate, summarize, and share data for team review and decision-making. The reliability and quality of the extracted data was also ensured through subsequent meetings, cross-checking of the collected data, discussions to resolve disagreement in data extraction, rereading of the full texts of the papers, refining the extraction form, and revising the collected data.

## Results

### Overview of Included Studies

**Figure 1:**
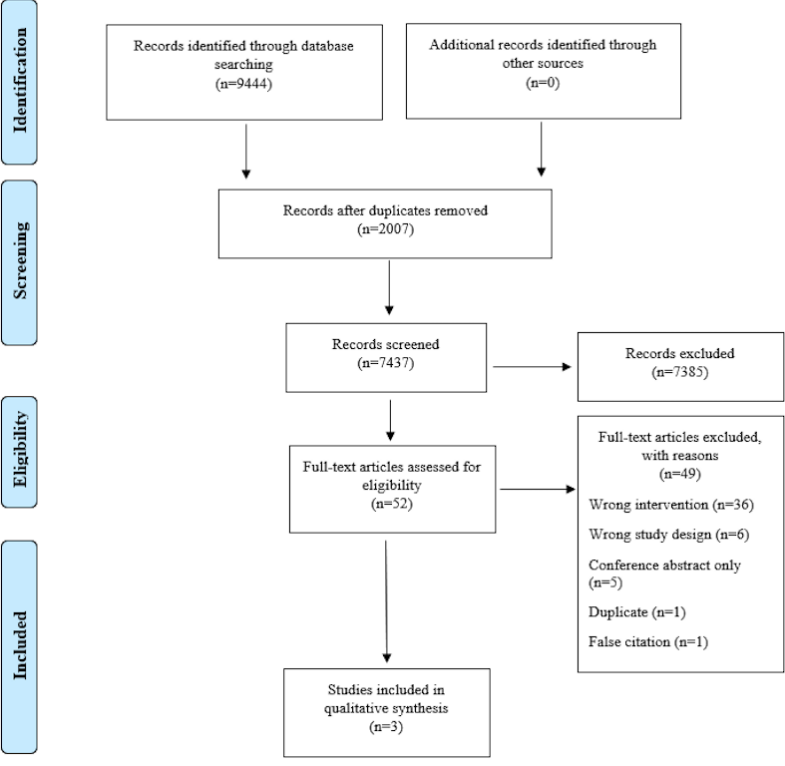
The PRISMA (Preferred Reporting Items for Systematic Reviews and Meta-Analyses) flow diagram.

Figure 1 depicts the PRISMA (Preferred Reporting Items for Systematic Reviews and Meta-Analyses) flow diagram. After duplicate removal, 7437 articles were individually screened; 52 full-text articles were screened for eligibility, and 36 articles were excluded because they were labelled as having the wrong intervention. These studies were closely examined by 2 independent reviewers (AF and DS) confirming they did not contain smartwatch technology and were therefore excluded for synthesis. Six studies were excluded due to wrong study design, as their overall objectives and approach was not to test smartwatch technology and also did not contain the right intervention. Five studies were excluded, as they were conference abstracts and full-text versions of these studies could not be retrieved from corresponding authors. The final 2 potentially eligible papers were excluded, as one was a duplicate and the other was a false citation. Ultimately, 3 studies were included for qualitative synthesis [37-39]. All included studies investigated a combined mHealth approach in participants with type 2 diabetes and included the use of a wearable device. Table 1 highlights the details of each study included. Some of the main findings from each study are presented in the next sections.

### Kim et al (2016)

In this paper [37], the authors introduced a patient-centerd smartphone–based diabetes care system (PSDCS) for patients with type 2 DM. They were instructed to use the PSDCS, which integrates a Bluetooth-connected glucometer, a digital food diary, and a wearable physical activity monitoring device. The primary end point was the change in HbA_1c_ from baseline after a 12-week intervention.

The application of the PSDCS to patients with inadequately controlled type 2 diabetes resulted in a significant HbA_1c_ reduction (from 7.7% to 7.1%) with tolerable safety profiles. There was no comment on the usability or durability of the intervention.

### Shaw et al (2020)

In this 6-month longitudinal feasibility study [38], the authors sought to examine the use of multiple mHealth technologies to generate and transmit data from diverse patients with type 2 DM in between clinic visits.

The study found that it was feasible for participants from different socioeconomic, educational, and racial backgrounds to use and track relevant diabetes-related data from multiple mHealth devices for at least six months. The study seemed to suggest that engagement with activity tools (eg, Fitbit technology) had the most success, while other technological engagements seemed to wane over time with some different demographic patterns in the engagement with these tools (eg, weight and glucose engagement tools).

### Zahedani et al (2021)

In this study [39], the authors sought to explore the potential benefit of CGM combined with a mobile app that links each individual’s glucose tracing to meal composition, heart rate, and physical activity in a cohort of people without diabetes and noninsulin- treated people with type 2 diabetes. The primary end point was the change in time in range (TIR), from the beginning to the end of a 10-day period of use of the FreeStyle Libre CGM.

Of those with suboptimal baseline TIR, 58.3% of participants with type 2 diabetes and 91.7% of healthy or prediabetes participants improved their TIR by an average of 22.7% and 23.2%, respectively. Predictors of improved response included no prior diagnosis of type 2 diabetes and lower BMI. There was no commentary on the usability or durability of the intervention.

## Discussion

### Principal Findings

In an increasingly technology-enabled world, strategies that harness the benefits of technology have the potential to address gaps in health care provision and address some of the most vexing clinical problems with concomitant improvements in the management of common chronic conditions across a broad population of patients. This scoping review sought to explore the evidence for the use of technology in the management of type 2 diabetes. The focus was on emerging yet increasingly available technologies in this field, such as smartphone apps and web applications, FGM, and smartwatch technology. Doupis et al [41] explored the peer-reviewed literature and found inconsistent benefits from applications that are automated for individualized feedback, such as Diabeo, Diabetes Pal and Blue Star with only Diabeo, using a telehealth-facilitated model showing benefits in HbA_1c_ reduction [41]. The addition of smartwatch technology to help change patient behaviors and enhance health literacy introduces the opportunity to explore real-time use of technology in this field. There is increasing interest from both the private healthcare industry and the technology sector as well as state sponsored providers due to the possibility of providing health care at scale with the continuously reducing cost of some of the technology. This scoping review focused on the use of FGM, as it is gaining momentum as an emerging technology in continuous glucose monitoring with early evidence of benefits in the management of type 2 diabetes [42-44]. Castellana et al [45], however, highlight in their meta-analysis that FGM did not show benefits in HbA_1c_ in patients when compared with traditional home-based glucose monitoring. There are several explanations for this outcome, including the lack of hypoglycemia and hyperglycemic alarms and a negative bias at low glucose concentrations, possibly resulting in the patient inadvertently adapting to higher glucose concentrations and thus higher HbA_1c_.

### Smartwatch and Its Potential to Support Diabetes Care

In 2020, 21% of Australians had a smartwatch and the wearables market is expected to grow by 14.5% annually from 2021 to 2026 [46]. Consumer smartwatches have grasped health research across a broad range of chronic diseases [34]. This scoping review highlights the limited studies that have explored the effect of smartwatch technology and its integration with continuous glucose monitoring in patents with type 2 diabetes. Most of the literature looking to integrate technology platforms has focused on popular lifestyle applications and associated technology, such as Fitbit [38]. The early studies by Kim et al [37] and Shaw et al [38] did not have control groups and had small sample sizes undermining the validity of the results. Zahedani et al [39] showed that the integration of data from FGM and a smartphone-based app is a feasible and multimodal data collection, with synthesis and feedback to participants provided by an mHealth app, and can significantly improve glycemic control, although the participants used the technology for only 10 days. Furthermore, the study is a nonrandomized observation study opening it to the risk of bias.

### Implications for Research

This scoping review clearly highlights the need for high quality studies exploring the effect of emerging technologies in an integrated fashion on the management of patients with type 2 diabetes. The research into the impact of both FGM and smartwatches, which are arguably more recent additions to the technological toolbox in health care provision for patients living with diabetes, needs further exploration.

### Implications for Practice

The provision of mHealth-supported, FGM-enhanced diabetic care can provide opportunities to improve health literacy and promote self-management for patients with type 2 DM and their treating teams through the data sharing of real-time glucose control. The impact of this and newer technological interventions such as web-based applications and mobile phone or smartphone apps that monitor a wide range of self-efficacy parameters need to be explored in a broader cohort of patients with type 2 diabetes. There is a great opportunity to influence health literacy, self-efficacy, and overall control of type 2 diabetes and its complications if these interventions can be delivered in a sustainable, cost-effective fashion.

### Limitations

There are several limitations to the use of emerging technologies in the management of diabetes. From the patient and clinician perspective, there are usability and affordability limitations to much of the proprietary technology available [47]. From a technology perspective, there is a need for accurate measurements of physiological parameters, full access to raw data in real time, and all technological tools on a compatible platform [48].

This scoping review sought to link the impact of these 3 technological developments as a bundle on behavior- and lifestyle-related diabetic self-management. Wu et al [49] showed that in patients with type 2 diabetes, mHealth apps can have a measurable impact on lifestyle modification, but this was measured mainly in regard to its impact on HbA_1c_ rather than other measures of self-efficacy and self-management behaviors [49]. Keller et al [50] showed that structured digital behavior change interventions infrequently have high-level evidence data to support their status as guideline base [50], and only one study by Quinn et al [51] showed a significant improvement in diabetic control in intervention versus the control group. As mentioned, the focus was mainly on HbA_1c_, and broader measures of health self-promotion were not measured. There seems to be a significant gap in the literature exploring the feasibility and usability of the use of the multipronged technological interventions and exploring the concept of technological fatigue in those whose condition is chronic, and thus, the interventions are expected to be lifelong.

### Conclusions

This scoping review highlights that there is scant peer-reviewed literature on the clinical impact of integrated emerging technologies used for the management of type 2 DM. As these technologies become more affordable, it is crucial that safe and validated digital health devices are increasingly available as part of the multimodal care for patients with type 2 diabetes. These emerging technologies have the potential to provide quantifiable and reliable data that can assist health professionals and hopefully prevent costly health complications. High-quality research needs to ensure that these interventions do not have unintended consequences of health care fatigue in an already at-risk population and that they deliver on the potential for improved control both in the short term and the longer term with the appreciation that diabetes is a chronic condition.

## Appendix

Multimedia Appendix 1 OVID Medline Search Strategy.

## Abbreviations

CGM: continuous glucose monitoring
DM: diabetes mellitus
FGM: flash glucose monitoring
mHealth: mobile health
PRISMA: Preferred Reporting Items for Systematic Reviews and Meta-Analyses
PSDCS: phone-based diabetes care system
RT-iCGM: real-time interstitial continuous glucose monitoring
TIR: time in range

## References

[1] Yoo E, Lee S (2010). Glucose biosensors: an overview of use in clinical practice. Sensors (Basel).

[2] Olczuk D, Priefer R (2018). A history of continuous glucose monitors (CGMs) in self-monitoring of diabetes mellitus. Diabetes Metab Syndr.

[3] Fokkert MJ, van Dijk PR, Edens MA, Abbes S, de Jong D, Slingerland RJ, Bilo HJG (2017). Performance of the FreeStyle Libre Flash glucose monitoring system in patients with type 1 and 2 diabetes mellitus. BMJ Open Diabetes Res Care.

[4] Twigg SM, Kazemi MR, Craig ME (2017). Flash continuous glucose monitoring and its IMPACT to REPLACE blood glucose monitoring in the management of type 1 and type 2 diabetes. US Endocrinol.

[5] Beck RW, Buckingham B, Miller K, Wolpert H, Xing D, Block JM, Chase HP, Hirsch I, Kollman C, Laffel L, Lawrence JM, Milaszewski K, Ruedy KJ, Tamborlane WV, Juvenile Diabetes Research Foundation Continuous Glucose Monitoring Study Group (2009). Factors predictive of use and of benefit from continuous glucose monitoring in type 1 diabetes. Diabetes Care.

[6] Wan W, Skandari MR, Minc A, Nathan AG, Winn A, Zarei P, O'Grady M, Huang ES (2018). Cost-effectiveness of continuous glucose monitoring for adults with type 1 diabetes compared with self-monitoring of blood glucose: The DIAMOND randomized trial. Diabetes Care.

[7] Hellmund R, Weitgasser R, Blissett D (2018). Cost calculation for a flash glucose monitoring system for UK adults with type 1 diabetes mellitus receiving intensive insulin treatment. Diabetes Res Clin Pract.

[8] Mancini G, Berioli M, Santi E, Rogari F, Toni G, Tascini G, Crispoldi R, Ceccarini G, Esposito S (2018). Flash glucose monitoring: a review of the literature with a special focus on type 1 diabetes. Nutrients.

[9] Raviteja KV, Kumar R, Dayal D, Sachdeva N (2019). Clinical efficacy of professional continuous glucose monitoring in improving glycemic control among children with type 1 diabetes mellitus: an open-label randomized control trial. Sci Rep.

[10] Bolinder J, Antuna R, Geelhoed-Duijvestijn P, Kröger J, Weitgasser R (2016). Novel glucose-sensing technology and hypoglycaemia in type 1 diabetes: a multicentre, non-masked, randomised controlled trial. The Lancet.

[11] Slattery D, Choudhary P (2017). Clinical use of continuous glucose monitoring in adults with type 1 diabetes. Diabetes Technol Ther.

[12] van Beers CAJ, DeVries JH, Kleijer SJ, Smits MM, Geelhoed-Duijvestijn PH, Kramer MHH, Diamant M, Snoek FJ, Serné EH (2016). Continuous glucose monitoring for patients with type 1 diabetes and impaired awareness of hypoglycaemia (IN CONTROL): a randomised, open-label, crossover trial. Lancet Diabetes Endocrinol.

[13] Balijepalli C, Druyts E, Siliman G, Joffres M, Thorlund K, Mills E (2017). Hypoglycemia: a review of definitions used in clinical trials evaluating antihyperglycemic drugs for diabetes. CLEP.

[14] Klonoff DC, Ahn D, Drincic A (2017). Continuous glucose monitoring: a review of the technology and clinical use. Diabetes Res Clin Pract.

[15] Khunti K, Alsifri S, Aronson R, Cigrovski Berković M, Enters-Weijnen C, Forsén T, Galstyan G, Geelhoed-Duijvestijn P, Goldfracht M, Gydesen H, Kapur R, Lalic N, Ludvik B, Moberg E, Pedersen-Bjergaard U, Ramachandran A, Investigator Group Hat (2016). Rates and predictors of hypoglycaemia in 27 585 people from 24 countries with insulin-treated type 1 and type 2 diabetes: the global HAT study. Diabetes Obes Metab.

[16] Briscoe VJ, Davis SN (2006). Hypoglycemia in type 1 and type 2 diabetes: physiology, pathophysiology, and management. Clin Diabetes.

[17] Duckworth W, Abraira C, Moritz T, Reda D, Emanuele N, Reaven PD, Zieve FJ, Marks J, Davis SN, Hayward R, Warren SR, Goldman S, McCarren M, Vitek ME, Henderson WG, Huang GD (2009). Glucose control and vascular complications in veterans with type 2 diabetes. N Engl J Med.

[18] Bae JP, Duan R, Fu H, Hoogwerf BJ (2017). Risk factors for nocturnal hypoglycemia in insulin-treated patients with type 2 diabetes: a secondary analysis of observational data derived from an integrated clinical trial database. Clin Ther.

[19] Gehlaut RR, Dogbey GY, Schwartz FL, Marling CR, Shubrook JH (2015). Hypoglycemia in type 2 diabetes--more common than you think: a continuous glucose monitoring study. J Diabetes Sci Technol.

[20] Kumareswaran K, Thabit H, Leelarathna L, Caldwell K, Elleri D, Allen JM, Nodale M, Wilinska ME, Evans ML, Hovorka R (2014). Feasibility of closed-loop insulin delivery in type 2 diabetes: a randomized controlled study. Diabetes Care.

[21] Thabit H, Hartnell S, Allen JM, Lake A, Wilinska ME, Ruan Y, Evans ML, Coll AP, Hovorka R (2017). Closed-loop insulin delivery in inpatients with type 2 diabetes: a randomised, parallel-group trial. Lancet Diabetes Endocrinol.

[22] Bally L, Gubler P, Thabit H, Hartnell S, Ruan Y, Wilinska ME, Evans ML, Semmo M, Vogt B, Coll AP, Stettler C, Hovorka R (2019). Fully closed-loop insulin delivery improves glucose control of inpatients with type 2 diabetes receiving hemodialysis. Kidney Int.

[23] Boughton CK, Bally L, Martignoni F, Hartnell S, Herzig D, Vogt A, Wertli MM, Wilinska ME, Evans ML, Coll AP, Stettler C, Hovorka R (2019). Fully closed-loop insulin delivery in inpatients receiving nutritional support: a two-centre, open-label, randomised controlled trial. Lancet Diabetes Endocrinol.

[24] Reddy N (2019). Meeting infant affect. Dev Psychol.

[25] Kalra S, Gupta Y (2015). Ambulatory glucose profile: flash glucose monitoring. J Pak Med Assoc.

[26] Matza LS, Stewart KD, Davies EW, Hellmund R, Polonsky WH, Kerr D (2017). Health state utilities associated with glucose monitoring devices. Value Health.

[27] Istepanian RSH, Al-Anzi TM (2018). m-Health interventions for diabetes remote monitoring and self management: clinical and compliance issues. mHealth.

[28] Bonoto BC, de Araújo VE, Godói IP, de Lemos LLP, Godman B, Bennie M, Diniz LM, Junior AAG (2017). Efficacy of mobile apps to support the care of patients with diabetes mellitus: a systematic review and meta-analysis of randomized controlled trials. JMIR Mhealth Uhealth.

[29] Brahmbhatt R, Niakan S, Saha N, Tewari A, Pirani A, Keshavjee N, Mugambi D, Alavi N, Keshavjee K (2017). Diabetes mHealth apps: designing for greater uptake. Stud Health Technol Inform.

[30] Veazie S, Winchell K, Gilbert J, Paynter R, Ivlev I, Eden KB, Nussbaum K, Weiskopf N, Guise J, Helfand M (2018). Rapid evidence review of mobile applications for self-management of diabetes. J Gen Intern Med.

[31] Piette J (2007). Interactive behavior change technology to support diabetes self-management: where do we stand?. Diabetes Care.

[32] Heintzman ND (2015). A digital ecosystem of diabetes data and technology: services, systems, and tools enabled by wearables, sensors, and apps. J Diabetes Sci Technol.

[33] Lu T, Fu C, Ma M, Fang C, Turner A (2017). Healthcare applications of smart watches. Appl Clin Inform.

[34] Reeder B, David A (2016). Health at hand: a systematic review of smart watch uses for health and wellness. J Biomed Inform.

[35] Munn Z, Peters MDJ, Stern C, Tufanaru C, McArthur A, Aromataris E (2018). Systematic review or scoping review? Guidance for authors when choosing between a systematic or scoping review approach. BMC Med Res Methodol.

[36] Arksey H, O'Malley L (2005). Scoping studies: towards a methodological framework. Int J Soc Res Methodol.

[37] Kim EK, Kwak SH, Baek S, Lee SL, Jang HC, Park KS, Cho YM (2016). Feasibility of a patient-centered, smartphone-based, diabetes care system: a pilot study. Diabetes Metab J.

[38] Shaw R, Yang Q, Barnes A, Hatch D, Crowley M, Vorderstrasse A, Vaughn J, Diane A, Lewinski A A, Jiang M, Stevenson J, Steinberg D (2020). Self-monitoring diabetes with multiple mobile health devices. J Am Med Inform Assoc.

[39] Dehghani Zahedani A, Shariat Torbaghan S, Rahili S, Karlin K, Scilley D, Thakkar R, Saberi M, Hashemi N, Perelman D, Aghaeepour N, McLaughlin T, Snyder MP (2021). Improvement in glucose regulation using a digital tracker and continuous glucose monitoring in healthy adults and those with type 2 diabetes. Diabetes Ther.

[40] Colquhoun HL, Levac D, O'Brien KK, Straus S, Tricco AC, Perrier L, Kastner M, Moher D (2014). Scoping reviews: time for clarity in definition, methods, and reporting. J Clin Epidemiol.

[41] Doupis J, Festas G, Tsilivigos C, Efthymiou V, Kokkinos A (2020). Smartphone-based technology in diabetes management. Diabetes Ther.

[42] Haak T, Hanaire H, Ajjan R, Hermanns N, Riveline J, Rayman G (2017). Flash glucose-sensing technology as a replacement for blood glucose monitoring for the management of insulin-treated type 2 diabetes: a multicenter, open-label randomized controlled trial. Diabetes Ther.

[43] Yaron M, Roitman E, Aharon-Hananel G, Landau Z, Ganz T, Yanuv I, Rozenberg A, Karp M, Ish-Shalom M, Singer J, Wainstein J, Raz I (2019). Effect of flash glucose monitoring technology on glycemic control and treatment satisfaction in patients with type 2 diabetes. Diabetes Care.

[44] Wada E, Onoue T, Kobayashi T, Handa T, Hayase A, Ito M, Furukawa M, Okuji T, Okada N, Iwama S, Sugiyama M, Tsunekawa T, Takagi H, Hagiwara D, Ito Y, Suga H, Banno R, Kuwatsuka Y, Ando M, Goto M, Arima H (2020). Flash glucose monitoring helps achieve better glycemic control than conventional self-monitoring of blood glucose in non-insulin-treated type 2 diabetes: a randomized controlled trial. BMJ Open Diabetes Res Care.

[45] Castellana M, Parisi C, Di Molfetta S, Di Gioia L, Natalicchio A, Perrini S, Cignarelli A, Laviola L, Giorgino F (2020). Efficacy and safety of flash glucose monitoring in patients with type 1 and type 2 diabetes: a systematic review and meta-analysis. BMJ Open Diabetes Res Care.

[46] SMART WEARABLE MARKET - GROWTH, TRENDS, COVID-19 IMPACT, AND FORECASTS (2022 - 2027) 2021. Mordor Intelligence.

[47] Walker A, Hood K, Gurka M, Filipp S, Anez-Zabala C, Cuttriss N, Haller MJ, Roque X, Naranjo D, Aulisio G, Addala A, Konopack J, Westen S, Yabut K, Mercado E, Look S, Fitzgerald B, Maizel J, Maahs DM (2021). Barriers to technology use and endocrinology care for underserved communities with type 1 diabetes. Diabetes Care.

[48] Schwartz FL, Marling CR, Bunescu RC (2018). The promise and perils of wearable physiological sensors for diabetes management. J Diabetes Sci Technol.

[49] Wu X, Guo X, Zhang Z (2019). The efficacy of mobile phone apps for lifestyle modification in diabetes: systematic review and meta-analysis. JMIR Mhealth Uhealth.

[50] Keller R, Hartmann S, Teepe GW, Lohse K, Alattas A, Tudor Car L, Müller-Riemenschneider F, von Wangenheim F, Mair JL, Kowatsch T (2022). Digital Behavior change interventions for the prevention and management of type 2 diabetes: systematic market analysis. J Med Internet Res.

[51] Quinn CC, Shardell M D, Terrin ML, Barr EA, Ballew SH, Gruber-Baldini AL (2011). Cluster-randomized trial of a mobile phone personalized behavioral intervention for blood glucose control. Diabetes Care.

