# Supplementary material for: The Clinical Impact of Flash Glucose Monitoring—a Digital Health App and Smartwatch Technology in Patients With Type 2 Diabetes: Scoping Review
Source: JMIR Diabetes. 2023 Mar 15;8:e42389. doi: 10.2196/42389 (PMC10131890; doi:10.2196/42389)
Supplement: Multimedia Appendix 1 [file diabetes_v8i1e42389_app1.docx]

OVID MEDLINE Search Strategy:

| **#** | **Searches** | **Results** |
| --- | --- | --- |
| 1 | Randomi#ed controlled trial.mp. | 566394 |
| 2 | Randomized Controlled Trial/ | 524407 |
| 3 | controlled clinical trial.mp. or Controlled Clinical Trial/ | 109108 |
| 4 | placebo.mp. | 222753 |
| 5 | Clinical Trial/ or trial.mp. | 1237776 |
| 6 | groups.mp. | 2301137 |
| 7 | Longitudinal Studies/ or longitudinal.mp. | 321309 |
| 8 | Cohort Studies/ or cohort.mp. | 699210 |
| 9 | 1 or 2 or 3 or 4 or 5 or 6 or 7 or 8 | 4003878 |
| 10 | smartphone.mp. or Smartphone/ or Cell Phone/ | 20844 |
| 11 | cell* phone.mp. | 10457 |
| 12 | mobile device.mp. or Computers, Handheld/ | 4910 |
| 13 | mobile application.mp. or Mobile Applications/ | 8204 |
| 14 | virtual reality.mp. or Virtual Reality/ | 12228 |
| 15 | online systems.mp. or Online Systems/ | 8561 |
| 16 | online.ti,ab,kw. | 131765 |
| 17 | Telemedicine/ or mhealth.mp. or m-health.mp. | 30631 |
| 18 | mobile health.mp. | 8725 |
| 19 | health, mobile.mp. | 124 |
| 20 | (telemedicine or mhealth or m-health or mobile health or health, mobile).ab,kw,ti. | 23490 |
| 21 | (interactive adj ((health adj communicat*) or video* or technolog* or multimedia)).ti,ab,kw. | 1311 |
| 22 | ((digital health or digital) adj3 intervention*).ti,ab,kw. | 1212 |
| 23 | ((mobile or cellular or cell or smart) adj (phone* or telephone*)).ti,ab,kw. | 13472 |
| 24 | patient monitoring device*.mp. | 40 |
| 25 | personal digital assistan*.mp. [mp=title, abstract, original title, name of substance word, subject heading word, floating sub-heading word, keyword heading word, organism supplementary concept word, protocol supplementary concept word, rare disease supplementary concept word, unique identifier, synonyms] | 1013 |
| 26 | wireless*.mp. | 16960 |
| 27 | (e-health or ehealth or electronic health).ti,ab,kw. | 25405 |
| 28 | Assist* technolog*.mp. [mp=title, abstract, original title, name of substance word, subject heading word, floating sub-heading word, keyword heading word, organism supplementary concept word, protocol supplementary concept word, rare disease supplementary concept word, unique identifier, synonyms] | 3088 |
| 29 | SMART technolog*.mp. [mp=title, abstract, original title, name of substance word, subject heading word, floating sub-heading word, keyword heading word, organism supplementary concept word, protocol supplementary concept word, rare disease supplementary concept word, unique identifier, synonyms] | 288 |
| 30 | SMART devic*.mp. [mp=title, abstract, original title, name of substance word, subject heading word, floating sub-heading word, keyword heading word, organism supplementary concept word, protocol supplementary concept word, rare disease supplementary concept word, unique identifier, synonyms] | 735 |
| 31 | platform.mp. | 142848 |
| 32 | (chat room* or chatroom*).ti,ab,kw. | 368 |
| 33 | app*.mp. | 6988908 |
| 34 | phone app*.mp. | 1244 |
| 35 | User-Computer Interface.mp. or User-Computer Interface/ | 38033 |
| 36 | human computer interaction*.ti,ab,kw. | 1213 |
| 37 | Internet.ti,ab,kw. | 56211 |
| 38 | (web base* or world wide web or WWW or website*).ti,ab,kw. | 63763 |
| 39 | Internet/ or IoT.mp. or "Internet of Things"/ | 77396 |
| 40 | internet of thing*.mp. | 2931 |
| 41 | telehealth.mp. | 6855 |
| 42 | remote sensing technology.mp. or Remote Sensing Technology/ | 3350 |
| 43 | Electronic Mail/ | 2743 |
| 44 | (Electronic Mail or e-mail*).mp. or email*.ti,ab,kw. [mp=title, abstract, original title, name of substance word, subject heading word, floating sub-heading word, keyword heading word, organism supplementary concept word, protocol supplementary concept word, rare disease supplementary concept word, unique identifier, synonyms] | 19418 |
| 45 | (Remote adj2 Monitoring).mp. [mp=title, abstract, original title, name of substance word, subject heading word, floating sub-heading word, keyword heading word, organism supplementary concept word, protocol supplementary concept word, rare disease supplementary concept word, unique identifier, synonyms] | 3480 |
| 46 | algorithm.mp. | 199230 |
| 47 | artificial intelligence.mp. or Artificial Intelligence/ | 33287 |
| 48 | machine learning.mp. or Machine Learning/ | 44316 |
| 49 | remote consultation.mp. or Remote Consultation/ | 5340 |
| 50 | 10 or 11 or 12 or 13 or 14 or 15 or 16 or 17 or 18 or 19 or 20 or 21 or 22 or 23 or 24 or 25 or 26 or 27 or 28 or 29 or 30 or 31 or 32 or 33 or 34 or 35 or 36 or 37 or 38 or 39 or 40 or 41 or 42 or 43 or 44 or 45 or 46 or 47 or 48 or 49 | 7394838 |
| 51 | 9 and 50 | 1175093 |
| 52 | diabet*.mp. [mp=title, abstract, original title, name of substance word, subject heading word, floating sub-heading word, keyword heading word, organism supplementary concept word, protocol supplementary concept word, rare disease supplementary concept word, unique identifier, synonyms] | 726132 |
| 53 | Diabetes Mellitus, Type 1/ or Diabetes, Gestational/ or Diabetes Mellitus/ or Diabetes Mellitus, Type 2/ | 324265 |
| 54 | (gestational diabetes or pregnancy induced diabetes).mp. [mp=title, abstract, original title, name of substance word, subject heading word, floating sub-heading word, keyword heading word, organism supplementary concept word, protocol supplementary concept word, rare disease supplementary concept word, unique identifier, synonyms] | 16186 |
| 55 | (type 1 diabetes or type one diabetes).mp. [mp=title, abstract, original title, name of substance word, subject heading word, floating sub-heading word, keyword heading word, organism supplementary concept word, protocol supplementary concept word, rare disease supplementary concept word, unique identifier, synonyms] | 42663 |
| 56 | (type two diabetes or type 2 diabetes).mp. [mp=title, abstract, original title, name of substance word, subject heading word, floating sub-heading word, keyword heading word, organism supplementary concept word, protocol supplementary concept word, rare disease supplementary concept word, unique identifier, synonyms] | 135599 |
| 57 | Glycated Hemoglobin A/ | 36196 |
| 58 | (Glycated Hemoglobin A or Glycosylated Hemoglobin A).mp. [mp=title, abstract, original title, name of substance word, subject heading word, floating sub-heading word, keyword heading word, organism supplementary concept word, protocol supplementary concept word, rare disease supplementary concept word, unique identifier, synonyms] | 36384 |
| 59 | HB A1c.mp. | 549 |
| 60 | HB A1c*.mp. | 550 |
| 61 | 52 or 53 or 54 or 55 or 56 or 57 or 58 or 59 or 60 | 729048 |
| 62 | 51 and 61 | 46285 |
| 63 | Blood Glucose Self-Monitoring/ | 7086 |
| 64 | blood glucose sens*.mp. [mp=title, abstract, original title, name of substance word, subject heading word, floating sub-heading word, keyword heading word, organism supplementary concept word, protocol supplementary concept word, rare disease supplementary concept word, unique identifier, synonyms] | 73 |
| 65 | blood glucose analysis.mp. [mp=title, abstract, original title, name of substance word, subject heading word, floating sub-heading word, keyword heading word, organism supplementary concept word, protocol supplementary concept word, rare disease supplementary concept word, unique identifier, synonyms] | 64 |
| 66 | (biosensing adj (technique or method)).mp. [mp=title, abstract, original title, name of substance word, subject heading word, floating sub-heading word, keyword heading word, organism supplementary concept word, protocol supplementary concept word, rare disease supplementary concept word, unique identifier, synonyms] | 161 |
| 67 | freestyle libre.mp. [mp=title, abstract, original title, name of substance word, subject heading word, floating sub-heading word, keyword heading word, organism supplementary concept word, protocol supplementary concept word, rare disease supplementary concept word, unique identifier, synonyms] | 210 |
| 68 | flash glucose monitor*.mp. | 328 |
| 69 | FSL.mp. | 1372 |
| 70 | interstitial glucose.mp. | 490 |
| 71 | (continuous glucose monitoring or CGM).mp. [mp=title, abstract, original title, name of substance word, subject heading word, floating sub-heading word, keyword heading word, organism supplementary concept word, protocol supplementary concept word, rare disease supplementary concept word, unique identifier, synonyms] | 5088 |
| 72 | blood glucose monitor*.mp. | 2156 |
| 73 | FGMS.mp. | 120 |
| 74 | 63 or 64 or 65 or 66 or 67 or 68 or 69 or 70 or 71 or 72 or 73 | 13714 |
| 75 | 62 and 74 | 1166 |
